# The Epidemiology and Control of Bovine Viral Diarrhoea Virus in Tropical Indonesian Cattle

**DOI:** 10.3390/pathogens11020215

**Published:** 2022-02-07

**Authors:** Widi Nugroho, Risma Juniarti Paulina Silitonga, Michael Philipp Reichel, Sri Handayani Irianingsih, Muhammad Satryo Wicaksono

**Affiliations:** 1 Laboratory of Veterinary Public Health, Faculty of Veterinary Medicine, Universitas Brawijaya, Malang 65151, Indonesia; muhammadsatryow@gmail.com; 2 Division of Laboratory Diagnostic Services, Center for Diagnostic Standards of Agriculture Quarantine, East Jakarta 13220, Indonesia; rismajps.21@gmail.com; 3 Department of Population Medicine and Diagnostic Sciences, Cornell University College of Veterinary Medicine, Ithaca, NY 14853, USA; mpr97@cornell.edu; 4 Disease Investigation Centre Wates, Kabupaten Kulon Progo, Wates 55602, Indonesia; shirianingsih@gmail.com

**Keywords:** bovine viral diarrhoea virus, control, smallholder, cattle, Indonesia

## Abstract

This review aims to update the knowledge of the epidemiology of Bovine viral diarrhoea virus (BVDV) in Indonesia and Southeast Asia and provide a perspective on the control options for BVDV in the Indonesian cattle population in the future. Studies on BVDV in Indonesia, since its first report in that country, and the updated beef and dairy cattle industries are reviewed. In ten of 34 provinces, BVDV is endemic. The subgenotypes of BVDV-1a and BVDV-1c are predominant in Indonesian cattle. However, BVDV is currently not a priority disease to control in Indonesia. Cattle imports from Australia appear to be potentially the most significant source of transmission of BVDV into native cattle, but the control of BVDV conducted in the local quarantine facilities is currently not achieving the aim of controlling BVDV; thus, complementary measures are needed. With the small-scale nature of the vast majority of cattle breeding in the country, the control of BVDV in provinces in which cattle breeding is economically essential may need to be organised by regional and provincial governments. Gaps in our knowledge of BVDV are identified in this review, and strategies for the control of BVDV in Indonesia are discussed.

## 1. Introduction

Bovine viral diarrhoea virus (BVDV) is a pathogen of cattle that manifests with various clinical manifestations. These may include abortion [1], congenital defects, the death of newborn calves [2], enteritis [3,4], respiratory disease, mortality [4], immunosuppression [5], emaciation [3], infertility [6] and mastitis [7].

BVDV belongs to the family of Flaviviridae, the genus Pestivirus, which recently has been reported to consist of 19 species, namely A to S [8]. Based on the analysis of the complete DNA sequence of the coding region, BVDV has a closer phylogenetic relation to Giraffe Pestivirus and HoBi-like virus of cattle and buffalo, than to two other essential livestock pestiviruses: border disease virus (BDV) of sheep and classical swine fever virus (CSFV) of pigs [9]. Two genotypes of BVDV are known: BVDV-1 and BVDV-2 [9]. BVDV-1 is Pestivirus A, and BVDV-2 is the Pestivirus B species in the taxonomy [8]. Analysis of the 5′UTR gene variability shows that BVDV-1 and BVDV-2 can be further separated into subgenotypes: twenty-one BVDV-1 (a–u) and four BVDV-2 (a–d). Subgenotypes BVDV-1a, BVDV-1b and BVDV-2a are the most prevalent worldwide [10]. BVDVs are also grouped into non-cytopathic (NCP) or cytopathic (CP) biotype groups based on their ability to produce defects in cell culture.

Persistently infected (PI) individuals are the most important source of infection for BVDV in cattle [11,12]. A PI may result when the dam, and subsequently the foetus in utero, is infected with BVDV during mid-gestation. Apart from producing PI, infection during gestation may also cause abortion or stillbirth. Unlike other infectious diseases, which shed virus temporarily over a short period, BVDV causes PI cattle to permanently shed a large amount of virus in their faeces and nasal fluids for life [13,14].

A PI animal may also develop mucosal disease, such as ulceration at the skin–horn junction, the interdigital skin, the pastern, dewclaws and the oral and nasal mucosa, linear ulcers in the upper alimentary tract, the rumen and the abomasum and diffuse thickening of the skin. In addition to mucosal disease, these animals may present with anorexia, sialorrhea, lameness and recumbency [15,16]. On the other hand, acutely infected (AI) calves might be able to shed virus into the environment through urine or nasal fluids for up to a month post-infection [4]. The classical method to detect and differentiate PI from AI is to perform a further test on positive animals after three or four weeks. All animals tested positive in both time points are considered PI. The test platforms available include virus isolation, ELISA and PCR [17,18].

Localised persistent infection of testicular tissue may occur in bulls that test negative for circulating virus yet shed virus persistently in their semen over a long period [19,20]. A persistently testicularly infected (PTI) bull did not transmit the virus to comingling bulls and steers [20]; however, transmission of BVDV may occur during insemination and may cause abortion [19,20].

BVDV may infect a wide range of domesticated ruminants other than cattle, including sheep, goats, buffalo and camel, as well as several wild ruminant species, including deer, ibex (*Capra pyrenaica*), giraffe (*Giraffa*), eland (*Taurotragus oryx*), wildebeest (*Connochaetes*), bushbuck (*Tragelaphus scriptus*), chamois (*Rupicapra Blainville*) and caribou (*Rangifer tarandus*) [9,21,22,23,24,25,26]. Buffalo, camel and many wild deer species have been considered accidental spill-over hosts [23]. BVDV has been known to infect pigs with mild to no clinical consequence. The virus was detected in tissues and the serum antibodies cross-reacted with classical swine fever virus (CSFV) [27]. Pigs may be persistently infected with BVDV, act as a lifelong reservoir for BVDV and shed virus into the environment [28].

BVDV infection causes economic losses in cattle industries worldwide due to production reduction and control costs. The magnitude of the economic impact of infection with BVDV on cattle production is varied, depending on the immune status of an animal and the virulence of the strain infecting the animal. The costs of BVD control include diagnostics, replacement, treatment of AI calves, biosecurity, disposal, increased labour, premature culling, costs of treatment of PI calves, vaccination, veterinary cost and diagnostics for the detection of PI and AI animals among young stocks [29]. In the cattle industries of western countries, the financial loss per animal in beef cattle is estimated to range from GBP 20.4 to GBP 56.1, while in dairy cattle it has been reported to range from GBP 0 to GBP 552 [29].

Various control methods have been proven effective at reducing the prevalence of or at eradicating BVDV from cattle populations. These controls include compulsory programs that combine PI detection and removal, movement restriction for infected herds, surveillance and strict biosecurity to prevent reinfection in free herds. These strategies have been successfully implemented with or without vaccination, but successful BVD control using a “vaccination only” program has never existed [14]. Economically speaking, there is no universal recommendation for preventative strategies in BVDV control, and selected strategies would depend on the herd and country-specific conditions [30].

The worldwide prevalence of PI calves, however, appears to have declined fivefold from 1.9% to 0.4% since 1980 at the animal level and decreased to nearly half, from 42.4% to 18.9%, at the herd level thanks to control programs implemented in western countries [31]. The prevalence of PI calves at the animal level is higher in countries that do not apply vaccination and control methods [31].

In Indonesia, BVD was reported for the first time in outbreaks of contagious diarrhoea during 1988 in five provinces—West and South Kalimantan, South Sulawesi, Lampung and Bengkulu provinces—with morbidity estimated to range from 0.23% to 30% and mortality from 0.02% to 90% [32,33]. In these outbreaks, watery diarrhoea, erosive lesion of the buccal mucosa, death due to dehydration and weakness were apparent. Imported cattle were suspected to be the source of infection [32]. The Indonesian government does not officially list BVDV as one of the priority animal diseases to control [34]. 

This review describes the cattle industries in Indonesia and the epidemiology of BVDV infection in Indonesian cattle and provides insight into the possible control options for the virus in the Indonesian cattle industries. A brief review of the epidemiology of BVDV in Southeast Asia is also presented to update on the topic in the region. For the purpose of this study, we searched articles from Google Scholar using the keywords “BVD”, “BVDV”, “BVDV” and “Indonesia”, “BVDV” and “Cambodia”, “BVDV” and “Lao-PDR”, “BVDV” and “Malaysia”, “BVDV” and “Myanmar”, “BVDV” and “Philippines”, “BVDV” and “Thailand”, “BVDV” and “Vietnam”, “BVDV” and “Singapore”, “BVDV” and “Brunei” and “BVDV” and “Timor-Leste”. We also obtained articles from the university repository and from personal communication with authors. In total, 28 references from Indonesian studies were collected, including 18 peer-reviewed research articles published in journals, one peer-reviewed review article published in a journal, and 9 non-peer-reviewed references including five theses, three conference proceedings, and one book. From Southeast Asia, nine peer-reviewed research articles published in journals were collected. The information on BVDV in Timor-Leste was obtained from an Indonesian study. All of these 37 references were included in this review.

## 2. A brief Description of the Cattle Industries in Indonesia

### 2.1. Beef Cattle Industry

**Figure 1:**
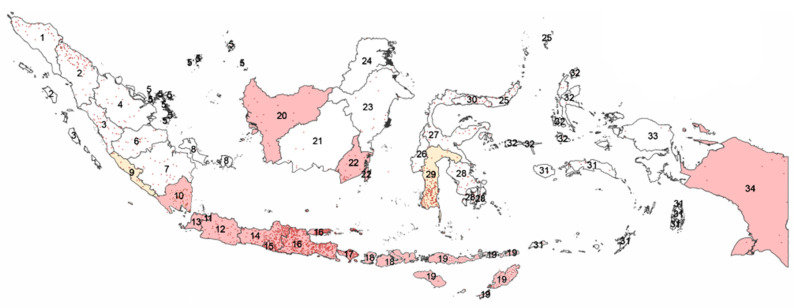
Distribution beef cattle population (red dots) in 2019 and Bovine viral diarrhoea virus (BVDV) endemic provinces (pink area = seroconversion and/or antigen detection; yellow area = suspected) by 2021 in Indonesia. Provinces: 1. Nanggroe Aceh Darussalam, 2. North Sumatra, 3. West Sumatra, 4. Riau, 5. Riau Islands, 6. Jambi, 7. South Sumatra, 8. Bangka Belitung, 9. Bengkulu, 10. Lampung, 11. DKI Jakarta, 12. West Java, 13. Banten, 14. Central Java, 15. DI Yogyakarta, 16. East Java, 17. Bali, 18. West Nusa Tenggara, 19. East Nusa Tenggara, 20. West Kalimantan, 21. Central Kalimantan, 22. South Kalimantan, 23. East Kalimantan, 24. North Kalimantan, 25. North Sulawesi, 26. West Sulawesi, 27. Central Sulawesi, 28. Southeast Sulawesi, 29. South Sulawesi, 30. Gorontalo, 31. Maluku, 32. North Maluku, 33. West Papua, 34. Papua [35,39,40,41,42,43,44,45,46,47,48,49,50].

By 2019, the total cattle population in Indonesia was estimated to be 17.7 million, with the total beef cattle population standing at 17.1 million [35]. As many as 60% of the beef cattle population resides in five provinces, with the largest cattle populations in the country being in East and Central Java, East and West Nusa Tenggara and South Sulawesi [35]. The distribution of the beef cattle population is shown in Figure 1. The eight areas with the densest cattle populations are the eastern part of North Sumatra, the southern part of Lampung, the eastern part of Central Java, Yogyakarta, the eastern part of East Java, Bali, the southern part of South Sulawesi and the southern part of East Nusa Tenggara province. With 50% of the country’s human population, Java Island contributes more than 70% of total beef consumption and 42.6% of the cattle population [35,36]. Smallholder beef farms comprise 99.96% of the national beef cattle farms, while only 0.04% of farms are owned by companies [37,38]. Small farms provide 65–70% of domestic beef production [36].

Beef cattle in Indonesia include native and exotic breeds. The native breed with the largest number and widest geographic distribution is Bali cattle [51]. Other native breeds are either descendants of Indian zebu, including Aceh, Pesisir and Sumba Ongole breeds, or crosses with Bali cattle and Indian zebu, including Madura, Jabres, Rancah, Rambon and Galekan cattle [51,52]. Exotic breeds include Ongole, Brahman, Brahman cross, Angus, Simmental, Limousine and Australian Commercial Cross [53]. The average small beef farm in Indonesia has 2.4 cattle, with the average size of household farms in East and Central Java being less than two animals [38,54,55], but, in Nusa Tenggara Barat, Nusa Tenggara Timur, Sulawesi, Maluku and Papua, the average size is more than three cattle [38]. As many as 76.2% of household farmers are breeders and mature female cattle comprise 45.7% of the total cattle population [38]. A village-level census in East Java reported that small-scale farms rely on artificial insemination for cattle breeding due to the lack of bulls, with the pregnancy rate reported to be just 26.8% [54]. As a result of low pregnancy and calving rates, domestic production can only satisfy 45% of the Indonesian demand for beef, and imports are needed to meet market demand [37].

Cattle operations in Indonesia involve importing cattle from Australia and transporting cattle amongst the islands of the entire country. A total of more than 1.1 million live cattle were imported into Indonesia through the Tanjung Priok seaport, Jakarta, throughout 2017–2020, with a total frequency of 824 shipments [56]. Importation has also occurred through the Lampung seaport at a volume of ~847 thousand cattle during the same period, with a total frequency of 672 shipments [56]. Cattle imports from Australia included feeders, finishers, female breeders and bulls [56] (Table 1).

**Figure 2:**
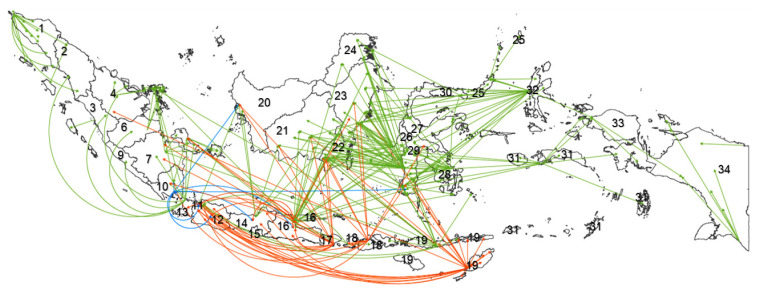
Cattle transport among provinces in Indonesia during 2019–2020. Green arrows represent total shipments of fewer than 75 thousand cattle, orange arrows represent shipments of 75–150 thousand cattle, and blue arrows represent shipments of more than 150 thousand cattle [56]. Provinces: 1. Nanggroe Aceh Darussalam, 2. North Sumatra, 3. West Sumatra, 4. Riau, 5. Riau Islands, 6. Jambi, 7. South Sumatra, 8. Bangka Belitung, 9. Bengkulu, 10. Lampung, 11. DKI Jakarta, 12. West Java, 13. Banten, 14. Central Java, 15. DI Yogyakarta, 16. East Java, 17. Bali, 18. West Nusa Tenggara Barat, 19. East Nusa Tenggara, 20. West Kalimantan, 21. Central Kalimantan, 22. South Kalimantan, 23. East Kalimantan, 24. North Kalimantan, 25. North Sulawesi, 26. West Sulawesi, 27. Central Sulawesi, 28. Southeast Sulawesi, 29. South Sulawesi, 30. Gorontalo, 31. Maluku, 32. North Maluku, 33. West Papua, 34. Papua.

The domestic transport of cattle is shown in Figure 2. Smaller total numbers of cattle (<75 thousand in total during the two years 2019–2020) were transported from South Sulawesi to South and East Kalimantan, from North Maluku to East Kalimantan and entire provinces in Sulawesi and from southern Maluku to Papua and Southeast Sulawesi. Transports of this size have also occurred from East Nusa Tenggara to South Kalimantan, East Kalimantan, South Sulawesi, Southeast Sulawesi and West Java; from East Java to East Kalimantan, South Kalimantan, South Sulawesi, Southeast Sulawesi and Riau Islands; and from West Java to the western part of Sumatra, Riau Islands and Bangka Belitung. Transports of smaller volumes of cattle have also occurred within Sumatra and Papua during the same period.

Intermediate numbers of cattle (75–150 thousand cattle) have been shipped from 2017 to 2020 from Bali to East and West Java, Bangka Belitung, South Sumatra and South, Central and West Kalimantan. West Nusa Tenggara transported medium numbers of cattle to South Sulawesi, East Kalimantan, South Kalimantan, East Java, West Java and Lampung. East Nusa Tenggara supplied medium numbers of cattle to Riau, entire provinces of Java, South, Central and East Kalimantan and South Sulawesi provinces. East Java provided medium numbers of cattle for East Kalimantan, Bangka Island, South Sumatra and Lampung. Larger totals of cattle (> 150 thousand heads) have been supplied from Lampung to West Kalimantan, Java and South Sulawesi during the years between 2017 and 2020 (Figure 2).

### 2.2. Dairy Industry

In 2013, a nationwide census counted 411.2 thousand dairy cows, owned by 142.0 thousand families, making the average size of a dairy farm 2.9 cows [38]; however, larger dairy farms also operate in Indonesia. Dairy cattle represent only around three per cent of the total cattle population in Indonesia.

In 2019, the dairy cattle population in Indonesia had increased to a number of 561.1 thousand, with milk production of 996.4 thousand metric tons [35]. Of this, 97.9% was produced in Java, and East Java alone contributes 52.5% of the national milk production [35]. However, the national production of dairy products is estimated to meet only 20% of domestic demand [57]. Apart from the national production, Indonesia imports skimmed milk powder from abroad, including from countries such as Australia, Belgium, Canada, Denmark, France, Germany, The Netherlands and New Zealand [56]. Currently, all cattle used for dairy production in the country are of the Friesian Holstein (FH) breed, and a few studies have reported the infection with BVDV [42,43,49]. The characteristics of dairy farming in Indonesia are similar to those of beef cattle farms, where replacement heifers are purchased at auction and only 22.1% of farmers produce their own replacement heifers [38]. Artificial insemination is the sole means of breeding on dairy farms. One of the differences between dairy farms and beef farms is that, while the beef farm system has no particular hierarchical organisation, dairy farms are much more organised in cooperatives, which subsequently supply most of their product to dairy companies [38].

## 3. The Epidemiology of BVDV in Indonesian Cattle Populations

### 3.1. Isolation and Detection

Indonesian strains of BVDV have been detected and isolated. Indonesian strains of non-cytophatic BVDV (NCP-BVDV) were successfully propagated in Madin-Darby Bovine Kidney (MDBK) cells and Bovine Turbinate Cell Lines (BTCL), and viruses were detectable after three days of culture, using immunoperoxidase staining [49,50,58,59,60]. However, the culture method may be prone to false-positive results, as commercial foetal bovine serum used to supplement the culture media may also be contaminated with BVDV [61]. ELISA immunoassays are a popular tool for detecting antibodies against or antigens of BVDV in Indonesian cattle [39,40,41,42,43,62,63], but, using p80 MoAb developed against a foreign strain of BVDV, only three of 12 NCP-BVDV Indonesian isolates were detectable in an immunoperoxidase monolayer assay test system [60]. This suggests that false-negative results may occur when surveys use imported p80-based immunoassays to detect BVDV [42].

Apart from culture- and protein-based tests, RNA-based tests have also been widely used to detect and characterise Indonesian isolates of BVDV, especially for genotyping [44,45,46,48,49].

### 3.2. Genetic Diversity

Studies have shown that the subgenotypes BVDV-1a, BVDV-1b and BVDV-1c circulate on Java Island of Indonesia (Table 2). Studies of BVDV subgenotypes in Indonesia mainly used genes NS5B or 5′UTR for analyses, but genes Npro, NS3 and E2 were also used. A study of a 360-nucleotide fragment of the NS5B gene of BVDV indicated that subgenotypes-1a, -1b and -1c were circulating in the Banyumas region, Central Java, during 2013–2016 [45]. The sequences used in this study are accessible in GenBank with the accession numbers MK411754, MK411755, MK411756, MK411757, MK411762, MK411764, MK411765, MK411761 and MK411759 for BVDV-1a, MK 411760 for BVDV-1b and MK411751, MK411763, MK411753, MK411758 and MK411752 for the BVDC-1c genotypes. Analyses of fragments of 5′UTR (275 nt), Npro (504 nt) and NS3 (2049 nt) and NS5B (1038–2157 nt) of BVDV isolates from Banyumas supported the finding that the subgenotype BVDV-1a is circulating in that region, while a recombination of BVDV-1a with BVDV-1c was indicated in an analysis of 1093 nt of the E2 gene [48]. The BVDV-1 genotype was also detectable in the goat population in Central Java in a study on 288 nt of the 5′UTR gene [44].

Subgenotypes BVDV-1a and BVDV-1c were detectable in a study of 360 nt fragments of the NS5B gene of BVDV isolates from the Malang and Pasuruan regions of East Java and isolates from the Cilacap, Boyolali and Semarang regions of Central Java [45]. The BVDV isolates of West Java were reported to belong to the genotype BVDV-1, when clustered using 360 nt of partial NS5B gene [46]. A study of BVDV genotyping in dairy cattle used samples from Java without describing the specific sample origin. Using the 5′UTR gene, the study detected BVDV-1a and BVDV-1c in dairy cattle from Java Island [49]. Genotyping of BVDV from other provinces of Indonesia has never been conducted. Additionally, a 288 nt length of the 5′UTR gene of BVDV isolated from East Java bovines was grouped into genotype BVDV-2, but, as the bootstrap value of the clustering was only 60%, this finding might be inconclusive [47].

### 3.3. Prevalence

The distribution of the BVDV endemic area in Indonesia by 2021 is depicted in Figure 1. Studies reported ten provinces to be endemic for BVDV in their cattle population and three provinces are suspected to be BVDV endemic. Provinces in Java are endemic areas, as many studies confirmed seroconversion and antigen detection [39,40,41,42,43,44,45,46]. Seroconversion to BVDV was also detected in cattle in Southern Papua, East Nusa Tenggara, West Nusa Tenggara Barat, Bali and Lampung [62,64,65]. Seroconversion of BVDV was detectable in an outbreak of cattle diarrhoea in West and South Kalimantan in 1989 [32,33]. The involvement of BVDV was suspected in diarrhoea outbreaks in 1989 in South Sulawesi and Bengkulu [32].

Prevalence studies of BVDV in Indonesia have been conducted using different methods. Therefore, prevalence estimates presented in this paper should be used with caution. The apparent seroprevalence of BVDV in Indonesian cattle populations ranges from 11.7–75.2% (Table 3), with seroprevalences reported from beef cattle in West Java at the highest end of this range (63.0–75.2%) between 2013 and 2016 [39,40,41]. Seroprevalence of BVDV in a dairy cow population in Yogyakarta, Central Java was lower, at 41.2–56.3% in the period between 2017 and 2020 [42,66]. Reported seroprevalences in beef cattle in provinces outside Java are much lower, ranging from just 0.3% (n = 108) in East Nusa Tenggara during the 1992 survey, 11.7% (n = 77) in Papua in 2017, 14.2% (n = 642) in West Nusa Tenggara in 1992, 34.8% (n = 161) in West Kalimantan in 1989 to 36.7% (n = 30) in Bali during 2020 [33,62,63,64]. In the Livestock Embryo Center (LEC) in Bogor, West Java, the seroprevalence of BVDV in diarrheic animals reached 48.8% (n = 43) in 2019 [43]. In the National Artificial Insemination Center (NAIC), BVD seroprevalence was 37.3% (n = 110) in 2011 [67]. The seroprevalence of BVDV in goats in Central Java was 10% (n = 20) in 2021 but was not detectable in sheep (n = 26) [44]. Infection with BVDV was detectable in cattle in East Java with the prevalence of 53.2% (n = 62) [45]. The prevalence of PIs in dairy cows in Java was as high as 6% (n = 200) in individuals with low reproductive performance, during 2018 [49].

### 3.4. Impact of BVD on Cattle Production in Indonesia

Investigations into the role of BVDV in diarrhoea outbreaks have been reported. The apparent risk of being BVDV antibody positive in an outbreak of bovine diarrhoea in South Kalimantan was estimated to be four times higher than that in an unaffected area, and co-infection with IBR (Infectious Bovine Rhinotracheitis) was detectable in some individuals [33]. The same study conducted in West Kalimantan showed that the association of BVDV with the outbreak of diarrhoea was not apparent [33]. In a recent study of diarrhoea and respiratory diseases of cattle in West and Central Java, the prevalence of BVDV, detected by RT-PCR targeting the NS5B gene was 11.7% (n = 588) of total clinical cases [46]. Furthermore, a case study at a Livestock Embryo Center (LEC) reported a high seroprevalence of BVDV in almost half of the diarrheic cattle tested [43]. In a study of diseases of pre-weaning calves in Central Lombok, Province of West Nusa Tenggara, diarrhoea dominated the clinical picture (42/57). However, only less than 2% (n = 42) of calves suffering from diarrhoea developed leukocytopenia, a possible indicator of viral disease [68]; the majority of sick calves in the study showed hypochromic anaemia (98.2%) and lymphocytosis (35.1%) [68]. These studies suggest that BVDV may play a role in diarrheic cases in Indonesian cattle, along with other causes.

In a cross-sectional survey of Bali breed cattle in Papua, infection with BVDV was associated with a threefold increased risk of being not pregnant [62]. The study in Java that revealed a PI prevalence of 6% in dairy cows with low reproductive performance [49] also indicated that the level of transient infection from PIs to other naive cows could have been high and caused reproductive problems. Several other bovine reproductive diseases are endemic in Indonesian cattle, including Infectious Bovine Rhinotracheitis, trichomoniasis, leptospirosis, bovine brucellosis, babesiosis, anaplasmosis and theileriosis [65,69,70,71]. Co-infections with BVDV and other reproductive pathogens in cattle were also reported [65]. These indicate that BVDV may have contributed to the lower reproductive performance of cows in Indonesia along with others of the abovementioned pathogens.

### 3.5. Risk Factors of Infection with BVD in Indonesian Cattle

A few studies have investigated the risk factors of infection with BVDV in Indonesian cattle populations. Seropositivity to BVDV in cattle was reported to increase with age [62,66]. The risk of being seropositive among Bali cattle in Papua was different between villages, and the difference was thought to be linked to transmission from other species, including wildlife [62]. Another study on dairy farms indicated that a larger farm size (more than four heads of cattle) was associated with seropositivity [66]. The latter might reflect a higher frequency of exposure of cattle to BVDV, such as cow replacement, insemination or trade-in cattle.

The risks of infection with BVDV due to poor farm biosecurity and untreated manure have been studied. One study reported that poor farm biosecurity, including unrestricted traffic of people and equipment onto a farm, the lack of isolation of cattle and dirty pens increased the risk of seroconversion to BVDV by three times, while the lack of manure management on a farm (which included three categories: low waste drain cleanliness, low frequency of pen cleaning and the lack of solid waste treatment by means of composting or combustion) was reported to increase the risk of BVDV seroconversion by a similar magnitude to that of the biosecurity factor [39]. That study was conducted on imported cattle from Australia at five quarantine facilities in West Java, and the cattle were not tested for BVDV before shipments. The fact that the prevalence of BVDV detected in this study was similar to that reported from the country of origin [39,40,41,72,73] may indicate that infections may have occurred before importation, and the statistically detectable risk factors may not relate to the situation in Indonesia.

## 4. BVDV in Southeast Asia (SEA)

Only a handful of studies are available on the epidemiology of BVDV in ruminants in SEA (Table 4). The reported seroprevalences of BVDV in cattle vary from as low as 6.4% (n = 471) in Cambodian cattle in 2016 [74] to 88.4% (n = 155) in cows of state-owned dairy farms in southern Vietnam in 2003 [75]. In the small-scale dairy farms of southern Vietnam, seroprevalence was low at 17.7% (n = 130), while the total seroprevalence of BVDV among dairy cows, including smallholder and large-scale farms, was 81.9% (n = 215) [75]. Further, the seroprevalence of BVDV in southern Vietnam was slightly higher in imported cows (88.4%, n = 155) compared to local cows (65.0%, n = 60) [75]. The reported seroprevalence of BVD was 45.7% (n = 1165) in north-eastern Thai dairy calves in 2011 [76]. 

In Selangor, Malaysia, the seroprevalence of BVDV on five dairy farms was reported to be 33.2% (n = 407), while individual seroprevalence among farms ranged from 0 to 75.9%, during the period from 2014 to 2015 [77]. In Lao PDR, the seroprevalence of BVDV was reported as 10.0% (n = 90) in 2013 [78]. In Myanmar, the herd-level seroprevalence of BVDV on small-scale dairy farms was low at 2.1% (n = 381) in 2016 [79]. A study in Luzon, the Philippines, in 2007 reported that 47.1% (n = 17) of aborting cows were RT-PCR-positive for BVDV. A survey in Timor-Leste in 1992 reported a BVDV seroprevalence of 9.3% (n = 108) [65]. BVDV was also reported to infect buffaloes in SEA. The seroprevalence of BVDV in buffaloes was low at 4.9% (n = 61) in Lao PDR, during 2013 [78] and 3.4% (n = 29) in Cambodia during 2016 [74]. In the Philippines, BVDV1b was reported in 2008 to infect water buffalo, but the prevalence was not reported [80]. 

Impacts of BVDV infection on cattle production were reported from the Philippines and Northeast Thailand, including abortion (OR: 27.11), longer calving intervals (OR: 1.29) and older age at first service (OR: 1.63) [76,81]. Risk factors associated with BVDV infection were reported from Lao PDR: being male (OR: 3.12, 1.22–7.99) and a higher *N. caninum* OD in the ELISA (OR: 1.87, 1.01–3.45) were two risk factors identified, while wet season (OR: 0.47, 0.24–0.93) and vaccination against foot-and-mouth disease or haemorrhagic septicaemia (OR: 0.91, 0.85–0.97) were thought to be preventative factors [82]. A study in the Philippines reported a proportion of 12.5% (n = 16) of bulls infected with BVDV, suggesting a possible risk of transmission via semen [81].

**Table 4:** Studies describing the epidemiology of Bovine viral diarrhoea virus (BVDV) in Southeast Asia ^a^.

Country	Period	Species	Test	Prevalence (n)	Risk Factors	Impact	Reference
Cambodia	2016	Buffalo	IDEXX BVDV Total Antibody Test Kit	3.4% (29)	-	-	[74]
Cambodia	2016	Cattle	IDEXX BVDV Total Antibody Test Kit	6.4% (471)	-	-	[74]
Malaysia, Selangor	2014–2015	Dairy cattle	PrioCHECK^®^ BVDV Ab, Prionics AG, Switzerland	33.2% (407)	-	-	[77]
Myanmar	2016	Dairy cattle (herd level)	Antibody detection, IDEXX	2.1% (381)	-	-	[79]
Southern Vietnam	2003	Dairy cattle	ELISA-kit (SVANOVA Biotech AB, Uppsala, Sweden)	81.9% (215)	-	-	[75]
Philippines	2008	Buffalo	RT-PCR: E2	- ^b^	-	-	[80]
Luzon, Philippines	2007	Brahman cows ^c^	RT-PCR: 5′UTR, E2	47.1% (17)	-	Abortion, OR: 27.11 (*p* < 0.001)	[81]
Luzon, Philippines	2007	Brahman bulls	RT-PCR: 5′UTR, E2	12.5% (16)	-	-	[81]
Northeast Thailand	2011	Dairy cattle with a high level of BTM ^d^ seroprevalence	Indirect ELISA kit, SVANOVIR^®^ BVDV-Ab (SVANOVA Biotech AB, Uppsala, Sweden)	36.1% (1165 young stocks)	-	Longer calving interval (OR = 1.29; *p* = 0.02) Older age at first service (OR = 1.63; *p* = 0.02).	[76]
Northeast Thailand	2011	Dairy cattle with a high level of BTM seroprevalence	ELISA BVDV-Ag test kit (IDEXX Laboratories B.V. Switzerland)	1.2% (1165 young stocks)	-	Longer calving interval (OR = 1.29; *p* = 0.02) Older age at first service (OR = 1.63; *p* = 0.02).	[76]
Lao PDR	2013	Buffalo	IDEXX BVDV Total Antibody Test Kit	4.9% (61)	-	-	[78]
Lao PDR	2013	Cattle	IDEXX BVDV Total Antibody Test Kit	10.0% (90)	-	-	[78]
Lao PDR	2016–2018	Cattle	IDEXX BVDV Total Antibody Test Kit	7.7% (390)	Male (OR: 3.12, 1.22–7.99) Wet season (OR: 0.47, 0.24–0.93) *N. caninum* OD ^e^ (OR: 1.87, 1.01–3.45) FMD/HS ^f^ vaccinated (OR: 0.91, 0.85–0.97)	-	[82]
Timor-Leste	1992	Cattle	Agar Gel Precipitation Antigen was supplied by Elizabeth Macarthur Agricultural Institute (EMAI, NSW, Australia)	9.3% (108)	-	-	[64]

^a^ BVDV has not been reported in two SEA countries: Brunei and Singapore. ^b^ BVDV1b was detected, but the prevalence was not reported. ^c^ Aborting cows. ^d^ BTM: Bulk Tank Milk. ^e^ *Neospora caninum* Optical Density in ELISA. ^f^ FMD: foot-and-mouth disease; HS: haemorrhagic septicaemia.

## 5. Control of BVD in Indonesian Cattle Population

Control strategies for BVDV in different sectors within the Indonesian cattle industry will need the central government to facilitate national regulation, supervision and incentives.

### 5.1. Vaccination

The development of a recombinant vaccine against BVDV in Indonesia using an adenovirus as a vector has been reported [83]. However, there is no commercial vaccine based on this research in Indonesia. The only BVD vaccine currently registered in Indonesia is an imported cocktail of modified live vaccines against BVD-IBR-Parainfluenza 3 and BRSV, produced by Novartis Animal Health Inc., US [84]. However, the efficacy of this vaccine under local conditions is unknown, but it is claimed to be safe for use in pregnant cows. 

### 5.2. Control of BVDV in the NAIC and LEC

In 2016, the central government of Indonesia launched a program aimed at increasing the cattle population by boosting the use of artificial insemination in the beef cattle population [37]. At the end of 2017, 92.3% of four million cows were reported to be artificially inseminated using semen from the NAIC [85]. The reported pregnancy rate was only 44.0% of the inseminated individuals, and the calving rate was 43.5% of the pregnancies or 19.1% of the reportedly inseminated cows [85]. The report of high levels of BVDV seroconversion in cattle at the NAIC suggests that contamination of semen used in the nationwide artificial insemination program with BVDV may be worthwhile to investigate among the possible factors that may contribute to the failure of the program [67]. 

The LEC conducts the production and distribution of cattle embryos in Indonesia. Annually, it produces and distributes 1200 embryos nationwide. It also produces bulls for breeding, and, by the end of 2020, it had distributed 399 bulls to provinces in Java, Sumatra, Kalimantan and Sulawesi islands [86]. The report that BVDV was prevalent in cattle at the LEC suggests a potential for transmission of BVDV nationwide through the distribution of PI bulls. Therefore, detection and removal of PI animals from both the NAIC and LEC appears prudent.

### 5.3. Control of BVDV in Internationally Imported Cattle

Currently, cattle are not tested for BVDV in the country of origin before shipment to Indonesia. According to Table 1, on average, 600–700 animals would have to be bled and tested daily, and, referring to the estimated PI prevalence in Australia of 1.4%, nine AI or PI animals might be expected to be detected daily in quarantine facilities. Moreover, during bull importation, ejaculates from 500 animals would have to be drawn and tested in the quarantine facilities to ensure that an imported bull is not carrying BVDV. For these reasons, quarantine offices should have enough personnel for blood sampling and ejaculate collections and sufficient laboratory capability for sample processing and testing.

Instead of using ELISA, PCR tests on pooled samples might be more practical, economical and time-saving, yet provide higher sensitivity than other methods for the detection of PI individuals [87,88]. Ear notch testing may offer a sampling methodology that is easier and faster than blood sampling, may be conducted by a non-specialised person or even farmers with simple training yet is capable of producing highly sensitive antigen detection results [89]. Furthermore, the pooling of ear notch samples for RT-PCR may accurately detect BVDV [89].

From our experience, some imported female cattle are pregnant and it is still possible that a PI calf might be born from this dam (the “Trojan” cow scenario) [90]. The birth of a PI calf might occur after a pregnant individual is released from a quarantine facility. Thus, it likely becomes another pathway for transmission of BVDV from imported cattle to local farms.

Currently, the available tests for BVDV in Indonesia for imported cattle are antigen and antibody ELISA, without semen tests to detect PTI. Further, only a small proportion of feeders are routinely bled and tested for BVDV antigen in quarantine. With a very low prevalence of PI, sampling techniques are likely not sensitive enough to detect and remove all PI or transiently infected individuals from imported cattle, and testing of all animals is needed. The seroprevalence of BVDV in the imported feeder and finisher cattle is reported to be high. Still, antigens have never been detected from serum samples of these animals in quarantine facilities [39,40,41].

Eliminating the risk of introducing AI or PI cattle to Indonesia cannot be assisted by selecting the source of feeder cattle for import strictly only from BVDV-free compartments within Australia as such compartments do not exist in that country. Therefore, the cattle have to be tested as free from BVDV before shipment. Alternatively, Indonesia might import only beef for domestic beef provision, but not live feeder cattle.

If live cattle imports were allowed only for breeding purposes but not for feeders, this would reduce the extensive workload for sampling in quarantine. Resources in quarantine facilities could be focused on performing more tests to detect PI. BVDV control during the importation of breeder bulls and heifers should include the requirement to purchase pre-tested non-PTI bulls and non-PI heifers from the country of origin and retest them for PI status upon arrival. Ideally, the heifers should not be pregnant before shipment to avoid the “Trojan” dam scenario. If pregnant cows are to be imported, the calves that they produce should be tested early in life to detect and remove PI individuals.

### 5.4. Control of BVDV during Cattle Transport among Islands within Indonesia

Due to currently ineffective controls of BVDV at the international borders, complementary mitigation strategies may need to be developed for the additional control of BVDV during cattle transport across the provinces to domestic quarantine facilities. For this purpose, an Indonesian province may be considered a zone, and three different zones might be proposed, according to domestic cattle movements. Lampung, Java, West Sulawesi, South Sulawesi, Southeast Sulawesi and North Maluku are importers and exporters; they may act as transit zones for cattle and diseases animals might carry. Kalimantan, Bangka Belitung and all the provinces in Sumatra except Lampung are end importer zones for cattle from Java. The eastern provinces, such as NTB, NTT and Bali, are sources of native cattle; they extensively export cattle to Sulawesi, Kalimantan, Java and Sumatra. These latter provinces are pure exporter zones (Figure 2).

Control of BVDV during cattle transport to end importer and transit zones should aim to protect local breeding farms. The level of BVDV control for this purpose could be varied among zones depending on the economic value of local breeding farms. For feeder cattle transported to regions within end importer and transit zones without any local breeding farms, the detection of BVDV during quarantine might not be necessary.

For end importer and transit zones with regions running breeding farms, all imported cattle should be tested negative for BVDV antigens before they are allowed to enter regions within these zones (a region is an administrative area within a province/zone; a province consists of several regions). Pregnant cattle (potential “Trojans”) should be banned from entering these zones. Vaccinations prior to mating could reduce the risk of the birth of PI calves [91,92]. Therefore, if pregnant cows were to be transported inter-regionally, they should have to be vaccinated before mating, test negative prior to shipment and be isolated at the destination until the calf delivery, and the offspring should have to be tested negative for BVDV before releasing to the general population. Further, imported feeder cattle should only be allowed to move straight to local abattoirs and not be allowed to enter live animal markets to avoid them comingling with local cattle. The regional government should provide compartments at the zone or regional borders for finishing or trading imported feeder or finisher cattle, separate from those for trading local cattle. For breeding, bulls should be monitored for PTI.

Local markets in a region may demand intact finisher bulls for slaughter during the Eid Al-Adha festivity, and traders may need to import finisher bulls from other zones. Antigen tests for these bulls should be required in zonal quarantine facilities unless all bulls are eventually slaughtered. Separation of imported from local bulls in the auction would not be needed as PI bulls would have been removed following the tests during the quarantine period. The antigen test in regional quarantine is beneficial, especially to anticipate situations when an imported bull is not sold for slaughter at the festival and needs to be sent to a farm within the region, comingle with local heifers or, due to their superior phenotype, tempts local farmers to purchase and use them for breeding with local heifers. 

For improved efficiency, the development of a capacity for BVDV testing in domestic quarantine facilities might be better co-organised by quarantine offices of adjoining zones that prioritise cattle breeding. In addition, the volume and frequency of cattle transportation into a zone should be used as one important consideration when the central government prioritises the development of domestic quarantine offices across the nation to prevent the intrusion of cattle diseases such as BVDV into a zone. 

As a complement to BVDV control during inter-zone transport, control of BVDV during cattle movement among regions within a zone should be developed. Some regions have erected inspection points for road-transported cattle at the regional border and require veterinary certificates as a prerequisite for cattle movement into a region. These regional cattle movements typically use trucks with a capacity of fewer than 20 cattle, but with high frequency. Using a rapid individual test for BVDV, such as the IDEXX SNAP^®^ BVDV Antigen Test, might aid the practicality of controlling BVDV at these transport inspection points. The sensitivity of the SNAP test to detect a local strain of BVDV warrants investigation.

### 5.5. Control of BVDV in Breeding Farms

The five provinces with the most cattle should prioritise controlling BVDV in their cattle breeding farms (Figure 1). With the distinct characteristic that most breeding farms in Indonesia are small scale [47,48], the BVDV control programs in these farms are likely to be practical when co-organised by regional and provincial governments. However, a successful control strategy for BVDV in breeding farms would depend on provincial or even region-specific conditions [30,93]. For example, reducing prevalence but not eradication was reported to be more economically sensible in Germany, and a simple method of an “ear tag testing and culling” strategy was the most preferred method [93]. On the other hand, a vaccination and biosecurity strategy was justified by a study in the UK [94].

Among the considerations for the choice of a suitable control strategy, factors to be considered are the type and size of farms, the prevalence of BVDV, the economic value of the cattle in a particular region, the economic impact of the disease and the estimated efficiency of a strategy [30]. The economic value of cattle production in Indonesia differs among provinces [38]. For example, the R/C (revenue/cost) ratio of cattle farming in East Nusa Tenggara was highest, and the R/C in South Sulawesi was lowest among the provinces in the country [38]. However, data such as BVDV prevalence, the impact of BVDV on cattle production, the cost of setting up a laboratory capacity, the cost of different test methods and farmers’ preferences on available control methods are largely lacking. Therefore, as part of a control program, studies are needed to provide those baseline data to assist the decision making for the selection of suitable methods that can reduce the economic impact of BVDV on the cattle industry in a particular region. Regional governments, which consider that local breeding farms are important, should be aware that many farmers purchase calves at auctions [38] and might allow them to comingle with cows on their farms. This practice may lead to transmission of BVDV from PI or AI animals onto the farm. Moreover, despite the extensive campaign of the central government for farmers to use artificial insemination during the last couple of years [37,85], local breeders might still be tempted to use a live bull for breeding due to its superior phenotype. In developing a laboratory capacity to control BVDV in breeding farms, a regional government should develop a program of bull health surveillance, along with routine tests for BVDV status and spot tests for weaned calves [87]. The capacity for development may also include monitoring the use of semen for artificial insemination and improving the hygienic practices of insemination. Further compartmentalisation at the sub-regional level might be necessary to contain the potential transmission of BVDV into a particular area.

### 5.6. Control of BVDV in Dairy Farms

Programs to control BVDV on dairy farms could be jointly organised by cooperatives and milk processors, with support from regional and provincial governments. As the farms are typically small, the program may be conducted at a village level, which usually contains ~200 cows, as the epidemiological unit. The program should include the detection and removal of PI calves. Incentive payments for calf removal for farmers may need to be shared by the cooperative, the company and the local government. Vaccination before insemination may be introduced to Indonesian dairy farms to prevent abortion or the birth of PI calves due to infection with BVDV in areas where the infection is prevalent.

### 5.7. Control of BVDV in Fattening Farms

In cattle-fattening productions in Indonesia that use only imported feeder cattle, studies indicate that the majority of individual cattle already have some level of immunity to BVDV. Thus, control of BVDV may not be warranted in these populations [39,40,41]. However, in local cattle-fattening operations, BVD seroprevalences are lower, with a significant proportion of naive and susceptible individuals [33,62,63]. The local fattening farm systems should be physically separated from the imported feeder farms. 

Fattening cattle mostly comingle with breeding cattle on small-scale breeding farms run by the same owner [38,54,55]. Purchasing grower cattle at auction and BVDV transmission from breeding animals may be likely sources of infection to fattening farms. Therefore, separating fattening farm cattle from the breeding cattle population is advisable. Testing of grower cattle before they enter onto a fattening farm could be offered to farmers who are keen to protect their farms from BVDV.

## 6. Conclusions

BVDV is endemic in 10 provinces in Indonesia; however, the prevalence differs between regions. The main BVDV subgenotypes circulating in Indonesia are BVDV-1a and BVDV-1c. The most likely sources of BVDV transmission in Indonesia are live cattle imported from Australia and contaminated semen from diseased bulls used in artificial insemination. The impact of this pathogen is most apparent on the reproductive performance of cows.

Control of BVDV in Indonesian cattle should include the detection and removal of BVDV infected individuals during the transport of live cattle from international seaports and between the islands in Indonesia and semen and bull production centres. To support the control of BVDV in small-scale cattle operations, the regional and provincial governments, which consider local cattle breeding farms economically significant, should determine suitable strategies for controlling BVDV in cattle populations. This needs to include developing a capacity to perform effective surveillance of PI animals. Further research is required in order to fully understand the economic impact BVDV has on the cattle industry in Indonesia.

## Figures and Tables

**Table 1:** Live cattle imports at Tanjung Priok and Lampung seaports between 2017 and 2020 [56].

Port	Number/Frequency	Feeder	Breeder Female	Breeder Bull	Total
Tanjung Priok	Number of cattle (n)	1,127,220	6603	2101	1,135,924
	Shipments (n)	796	24	4	824
Lampung	Number of cattle (n)	843,328	3553	-	846,881
	Shipments (n)	658	14	-	672

**Table 2:** Studies describing the genetic diversity of Bovine viral diarrhoea virus (BVDV) in Indonesian cattle and goat populations.

No.	Province	Species	Region	Gene	Nucleotide Length	Result	Reference
1	East Java	Cattle	Pasuruan	NS5B	360	BVDV-1c	[45]
			Malang	NS5B	360	BVDV-1a	[45]
			Ngawi	5′UTR	288	BVDV-2	[47]
2	Central Java	Cattle	Boyolali	NS5B	360	BVDV-1a	[45]
			Cilacap	NS5B	360	BVDV-1c	[45]
			Semarang	NS5B	360	BVDV-1c	[45]
			NS *	NS5B	360	BVDV-1	[46]
			Banyumas	NS5B	360, 1038, 2157	BVDV-1a, BVDV-1b, BVDV-1c	[45,48]
			Banyumas	5′UTR	275	BVDV-1a	[48]
			Banyumas	NPro	504	BVDV-1a	[48]
			Banyumas	NS3	2049	BVDV-1a	[48]
			Banyumas	E2	1093, 1122	BVDV-1c	[48]
		Goats	NS	5′UTR	288	BVDV-1	[44]
3	Jakarta	Cattle	NS	NS5B	360	BVDV-1	[46]
4	West Java	Cattle	Pengalengan, Lembang, Bogor, Sumedang	NS5B	360	BVDV-1	[46]
5	Java	Dairy Cattle	NS	5′UTR	288	BVDV-1a, BVDV-1c	[49]

* NS = Not Specified

**Table 3:** Studies describing the prevalence, risk factors and impact of infection with Bovine viral diarrhoea virus (BVDV) to the cattle industry in Indonesia.

Province	Region	Period	Breed	Method of Diagnosis	n	Prevalence	Risk Factors	Impact	Reference
Papua	Timika	2017	Bali cattle	ELISA Ab & Ag (IDEXX BVD Total Antibody Test kit, IDEXX HerdChek BVD Ag/Serum Plus Test kit)	77	11.7%	Different village RR: 7.94 (1.01–62.27)	Pregnancy failure RR: 2.9 (1.02–8.14)	[62]
West Kalimantan	NS ****	1989	NS	Serum Neutralisation Test (SNT)	161	34.8%	Possibly co-infection with IBR ***	Diarrhoea RR: 1.3 (1.2–1.5)	[33]
East Nusa Tenggara	Sikka, Kupang, Sumba Timur, Sumba Barat	1992		Agar Gel Precipitation Antigen was supplied by Elizabeth Macarthur Agricultural Institute (EMAI, NSW, Australia)	320	0.3%	ND **	ND	[64]
West Nusa Tenggara	Lombok Tengah, Lombok Timur, Lombok Barat, Sumbawa Besar, Bima, Dompu.	1992		Agar Gel Precipitation Antigen was supplied by Elizabeth Macarthur Agricultural Institute (EMAI, NSW, Australia)	642	14.2%	ND	ND	[64]
Bali	Badung, Gianyar, Bangli, Klungkung, Karangasem, Buleleng, Jembrana, Tabanan.	1992		Agar Gel Precipitation Antigen was supplied by Elizabeth Macarthur Agricultural Institute (EMAI, NSW, Australia)	682	13.5%	ND	ND	[64]
Bali	Gianyar, Badung	2020	Bali cattle	ELISA Ab (NS)	30	36.7%	ND **	ND	[63]
East Java	Pasuruan, Batu	2019-2020	NS	ELISA Ab (IDEXX BVDV Total Ab Test, Switzerland)	62	53.2%	ND	ND	[65]
Yogyakarta	Sleman	2017	FH *	(ID Screen^®^ BVD p80 Antibody Competition, IDvet France), (IDEXX BVDV Ag/Serum Plus^®^, IDEXX, US)	255	41.2% ^SS^	Older age RR: 1.01 (CI: 1.02–1.04) Larger farm size RR:1.11 (CI: 1.01–1.22)	ND	[66]
Yogyakarta	Sleman	2020	FH	ELISA Ab (ID Screen^®^ BVD p80 Antibody Competition, IDvet France)	96	56.3%	Manure for Biog RR:1.4 (CI: 1.0–2.0) Clean pen RR: 1.5 (CI: 0.7–3.2)	ND	[42]
West Java	Cianjur, Bogor, Tangerang, Bandung, Subang.	2016	Brahman cross	ELISA Ab (IDEXX, NS)	100 ^0^	63.0%	Biosecurity ^1^ RR: 3.3 (CI: 1.4–8.0) Manure management ^2^ RR:2.7 (CI: 1.1–6.4)	ND	[39]
West Java	Cianjur, Bogor, Bandung, Subang	2015	Brahman cross	ELISA Ab (IDEXX-BGVV B233)	474	67.5%	ND	ND	[40]
West Java	Livestock Embryo Center, Bogor	2019	FH, Ongole, Limousine, Simental, Angus, Wagyu	ELISA Ab (IDEXX, NS)	43 ^3^	48.8%	ND	ND	[43]
West Java	West Bandung, Bogor	2019–2020	NS	ELISA Ab (IDEXX BVDV Total Ab Test, Switzerland)	47	46.8%	ND	ND	[65]
Banten	Legok, Tangerang	2013	Brahman cross	ELISA Ab A (IDEXX-BGVVB233)	230	75.2%	ND	ND	[41]
Java	National Artificial Insemination Center	2011	NS	NS	110	37.3%	ND	ND	[67]
Java	NS	2018	FH	RT-PCR (5′UTR)	200 ^4^	6% ^5^	ND	ND	[49]
Lampung	NS	2019–2020	NS	ELISA Ab (IDEXX BVDV Total Ab Test, Switzerland)	18	11.0%	ND	ND	[65]

**** NS = Not Specified. *** IBR = Infectious Bovine Rhinotracheitis. ** ND = Not Determined. * FH = Friesian Holstein. ^0^ The study was conducted in quarantine facilities; subjects were imported cattle. ^1^ Included: isolation of incoming animals, restriction of visitors and equipment, pen cleanliness. ^2^ Included: drainage cleanliness, pen cleaning frequency, manure treatment. ^3^ Sampling technique was purposive; samples were chosen from individuals that showed low body scores, nasal discharge or diarrheic. ^4^ Samples were dairy cattle that showed low reproductive performance. ^5^ Prevalence of Persistently Infected (PI) animals. ^SS^ The prevalence was 100% at the farm level (n = 63).

## Data Availability

Not applicable.
